# Micro/nanorobotics for sepsis diagnosis and therapy: a frontier in precision medicine

**DOI:** 10.7150/thno.139332

**Published:** 2026-08-12

**Authors:** Yukun Liu, Kang Wang, Guoyun Wan, Fangli Gao, Huiming Tang, Xiangjun Bai, Zhanfei Li, Yuchang Wang

**Affiliations:** 1 Department of Plastic and Aesthetic Surgery, Tongji Hospital, Tongji Medical College, Huazhong University of Science and Technology, Wuhan, 430030, China.; 2 School of Life Science and Technology, Henan Medical University, Xinxiang, 453003, China.; 3 College of Chemistry and Chemical Engineering, Henan Normal University, Xinxiang, 4453007, China.; 4 Institute of Pharmacology and Clinical Pharmacy, Goethe University Frankfurt, 60438 Frankfurt am Main, Germany.; 5 Division of Trauma Surgery, Tongji Hospital, Tongji Medical College, Huazhong University of Science and Technology, Wuhan, 430030, China.; 6 Sino-German Research Institute of Disaster Medicine, Huazhong University of Science and Technology, Wuhan 430030, China.; 7 Department of Emergency and Critical Care Medicine, Tongji Hospital, Tongji Medical College, Huazhong University of Science and Technology, Wuhan, 430030, China.

**Keywords:** micro/nanorobots, sepsis, therapy, drug delivery, nanomedicine

## Abstract

Sepsis is a serious, life-threatening widespread inflammatory response in the body to infection that can result in multiple organ damage and has a relatively high death rate. Traditional antibiotics and supportive treatments have certain deficiencies, such as poor drug accumulation at the site of infection, delayed identification of pathogens, and an inability to precisely regulate the host immune response. New developments in micro- and nanorobotics have provided new paths for sepsis diagnosis and treatment by means of active motion, intelligent sensing and targeted intervention. Biohybrid microrobots containing DNA nanodevices or enzyme sensors can be used for diagnosis to rapidly detect bacterial pathogens, endotoxins and other markers such as procalcitonin. Motivated micro-robots can overcome the biofilm barrier to deliver drugs and increase the local concentration of antibiotics to inhibit bacterial toxins. Functionalised nanobots of cell membrane or enzyme types can target cytokines, reactive oxygen species and circulating mitochondrial DNA to suppress inflammation and organ damage. The above-mentioned multi-functional platforms have combined pathogen clearance, immune modulation and tissue repair into one treatment. Although the above are promising, there are still some problems in biosafety, propulsion efficiency under complex physiological conditions, large-scale production, and the development of clinically relevant animal models. Future work will focus on building intelligent, biocompatible systems capable of autonomous navigation and adaptation to sepsis. Micro- and nanorobots are generally considered to be a new type of devices for treating sepsis that can achieve targeted and personalised therapy through intelligence.

## 1. Introduction

Sepsis is a serious disease caused by infection, which involves an abnormal response of the host, often leads to multiple organ failure and has a high death rate 1. According to the Global Burden of Disease Study 2017, there were about 48.9 million cases of sepsis and more than 11 million sepsis deaths worldwide in 2017, making up almost 20 per cent of all deaths globally 2. Although there have been recent developments in critical care medicine, sepsis is still a serious problem for public health around the world because of its numerous causes and rapid progression. At present, there are no dedicated methods for the detection and treatment of this disease in time. Only in time can we identify and manage the problem of sepsis early 3. At present, most diagnostic methods have been limited by non-specific clinical signs and a lack of sensitive and specific biomarkers 4-7. Early detection of sepsis by the traditional markers procalcitonin (PCT), C-reactive protein (CRP) and interleukin-6 (IL-6) is generally inaccurate 4-6. Microbial cultures are commonly used to identify pathogens, but it may take as long as 72 hours. It has a relatively high false-negative rate and may thus fail to diagnose timely serious diseases, causing harm to life. Broad-spectrum antibiotics are still used to treat sepsis in a general way; however, due to the spread of antimicrobial resistance around the world, they are becoming less effective^7^-^8^. Empirical or excessive antibiotic use will also cause harm to health by promoting the development of multidrug-resistant organisms and dysbiosis 9, 10. Furthermore, sepsis often presents with a severe immune dysregulation characterized by both hyperinflammation and immunosuppression, and effective immunomodulatory therapy has yet to be developed 11. Collectively, the above deficiencies indicate that new platforms with fast pathogen detection, precise antimicrobial therapy and immune system regulation are urgently needed.

Recently, Nanomedicine has begun to develop as a new way of handling 12-16. Many nanotechnologies have been introduced in recent years, such as liposomes, polymeric nanoparticles, metal-organic frameworks (MOFs), nanozymes and nanoscale biosensors, all for the purpose of targeted drug delivery, biomarker recognition and immunomodulation 3, 14, 17, 18. The following are the advantages of nanosystems over the traditional method: improved drug solubility; extended systemic circulation time; and enhanced extravasation and retention via the enhanced permeability and retention (EPR) effect 19-20. Most of these systems are passive-targeting approaches 21 and thus less effective in dealing with the complex multifactorial pathophysiology of sepsis, such as infection, cytokine storms and immunosuppression 19.

To overcome the above shortcomings, micro- and nanorobots have gained considerable attention as intelligent, self-propelled, multifunctional nanoscale platforms for biomedical applications 20-22. These robots are generally a few hundred nanometers to several micrometers in size and can move autonomously by means of chemical catalysis, external magnetic fields, ultrasound, light or biological motors 20-23. Because they are mobile, these organisms can reach the interior of blood vessels and bacterial biofilms in the body and thus avoid being blocked by passive nanoparticles 24. Micro/nano-robots can be equipped with various functional payloads, such as antibiotics, anti-inflammatory drugs, immunomodulatory agents and imaging probes, and thus achieve spatiotemporal control of release at the site of infection or inflammation 25-27. These platforms provide a good opportunity for integrated "theranostic" systems that combine real-time biomarker detection, in situ monitoring of infection severity, and precise, on-demand therapeutic intervention in a single device 28. Micro/nano-robots are more adaptable, have higher targeting accuracy and are multi-functional compared with traditional nanomedicines 29.

Although they have good prospects, there are no all-encompassing reviews of the applications of micro- and nanorobots in sepsis diagnosis and treatment. Most of the existing research has focused on traditional nanoplatforms or cancer-related applications, and the specific engineering strategies, application scenarios and translational challenges of micro/nanorobots in sepsis have not been fully explored 30. Based on the above knowledge deficiency, this paper will systematically examine the latest developments in micro- and nanorobotic systems for sepsis treatment. First, the pathogenesis of sepsis is introduced, and then the Design ideas, driving mechanisms and functionalisation strategies for micro- and nanorobots are presented. Then, we will introduce how they are used for pathogen detection, monitoring the inflammatory microenvironment, targeted antimicrobial therapy and immune modulation. Finally, we will introduce some of the problems that need to be solved before these intelligent and multi-functional micro/nanorobots can be used in clinical practice, such as biocompatibility, *in vivo* stability, and toxicity, and offer some directions for future research.

## 2. Pathophysiology and Therapeutic Challenges of Sepsis

### 2.1 Pathophysiological Mechanisms of Sepsis

Sepsis is a relatively unstable pathological condition caused by an abnormal response of the host immune system to infection; it presents as a state of both hyperinflammation and immunosuppression 31-33. The innate immune system will rapidly recognise PAMPs (pathogen-associated molecular patterns) on pathogens, such as lipopolysaccharide (LPS) of Gram-negative bacteria (e.g., Escherichia coli and Pseudomonas aeruginosa), lipoteichoic acid from Gram-positive bacteria (e.g., Staphylococcus aureus and Streptococcus species), and β-glucan of fungi (e.g., Candida albicans), and initiate a response. These PAMPs are recognised by pattern recognition receptors (PRRs), such as Toll-like receptors (TLRs) and NOD-like receptors (NLRs), expressed on macrophages, dendritic cells, and other immune cells; thus, a signalling cascade is activated, and pro-inflammatory cytokines such as TNF-α, IL-1β and IL-6 are robustly released 34-36. Therefore, a "cytokine storm" occurs rapidly to amplify inflammation; at the same time, endothelial cells become activated and dysfunctional, increased vascular permeability results, and coagulation disorders occur 37, 38. Disseminated intravascular coagulation (DIC) and extensive microthrombus formation have also reduced blood supply to the tissues and caused hypoxia 39, 40.

**Figure 1:**
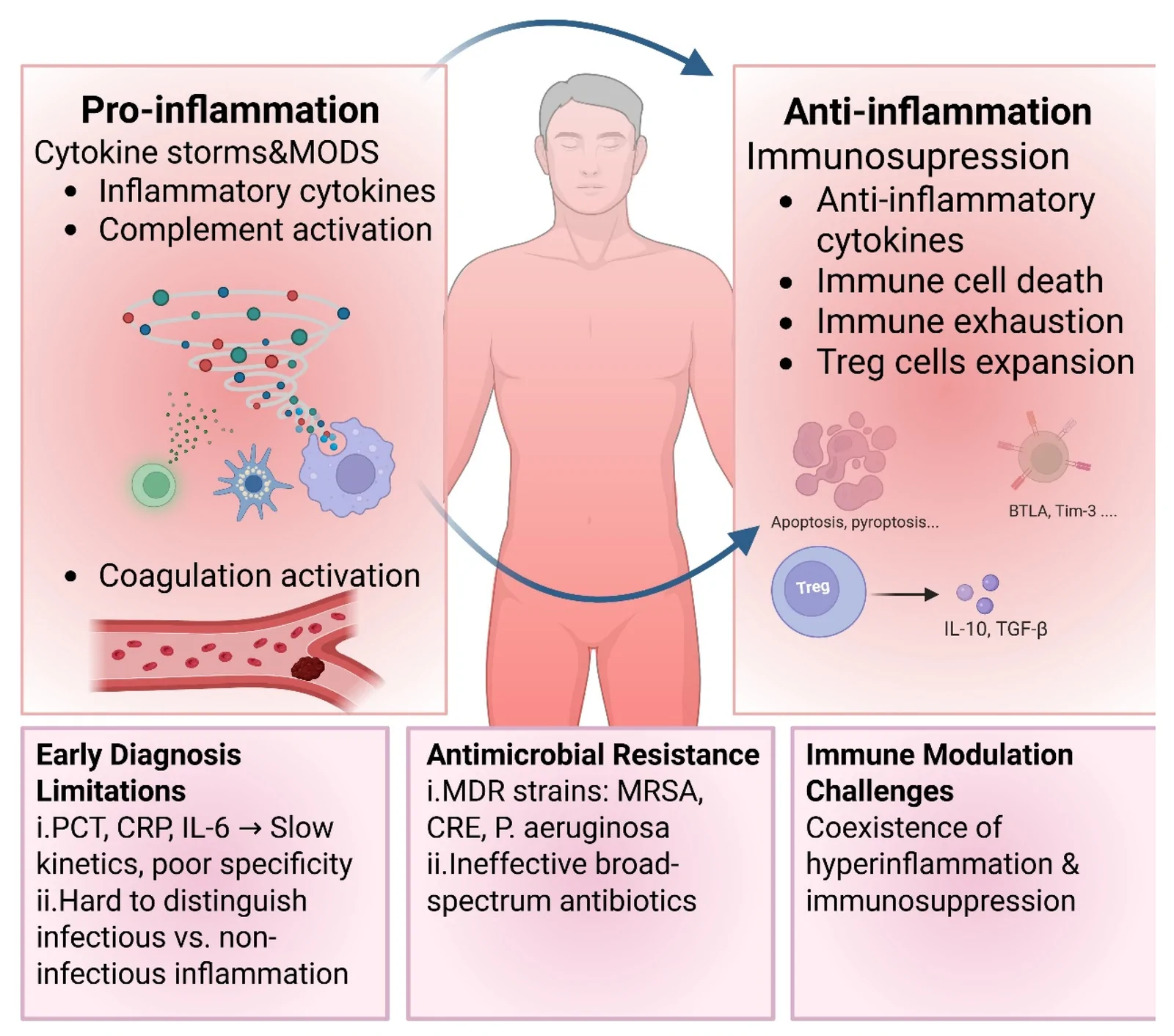
Schematic representation of the dynamic balance between pro-inflammatory and anti-inflammatory responses in systemic inflammation. The pro-inflammatory stage is a cytokine storm, complement activation and coagulation activation; thus, there is tissue damage and multiple organ dysfunction syndrome (MODS). On the other hand, the anti-inflammatory stage is associated with immunosuppression, an increase in anti-inflammatory cytokines, immune cell death and exhaustion, and an expansion of regulatory T-cells (Tregs). Coexistence and cyclical oscillations of hyperinflammation and immunosuppression further intensify the disorder of the whole-body immune system. Based on the clinical disease background, many different diseases have occurred; therefore, personalised intelligent medical care should be applied (Created using Biorender.com).

Notably, this hyperinflammatory phase often occurs at the same time as or after significant immunosuppression, with increased apoptosis of T and B lymphocytes, monocyte dysfunction, expansion of regulatory T cells (Tregs), and elevated levels of anti-inflammatory cytokines such as IL-10 and TGF-β 32, 41. Immune cells are exhausted at this time, so they cannot fight off pathogens effectively and are therefore more likely to be re-infected 42-44. Recently, many studies have also shown that immunometabolic reprogramming is required for the differentiation of immune cells, and at the same time, alterations in metabolic pathways, such as glycolysis and oxidative phosphorylation, can either promote or inhibit inflammation 45-46. The "biphasic" immune dysregulation thus establishes a vicious cycle: prolonged inflammation damages host tissues, immune suppression increases susceptibility to secondary infections, and thereby further advances the disease 47-48. The above pathological changes occur in a systemic manner and lead to multi-organ dysfunction syndrome (MODS), which is responsible for the high mortality rate of sepsis 49. Organ-specific expressions are very serious. Disruption of the capillary barrier in the lungs causes acute respiratory distress syndrome (ARDS). Renal ischaemia and inflammation cause acute kidney injury (AKI). Liver disease may present as cholestasis and metabolic disorders. Cardiovascular compromise occurs due to myocardial depression and loss of vascular tone, resulting in a reduced cardiac output and circulatory failure 50, 51. Mortality will rise as the organs gradually fail due to delayed or absent treatment (Figure 1).

### 2.2 Current Problems

**Figure 2:**
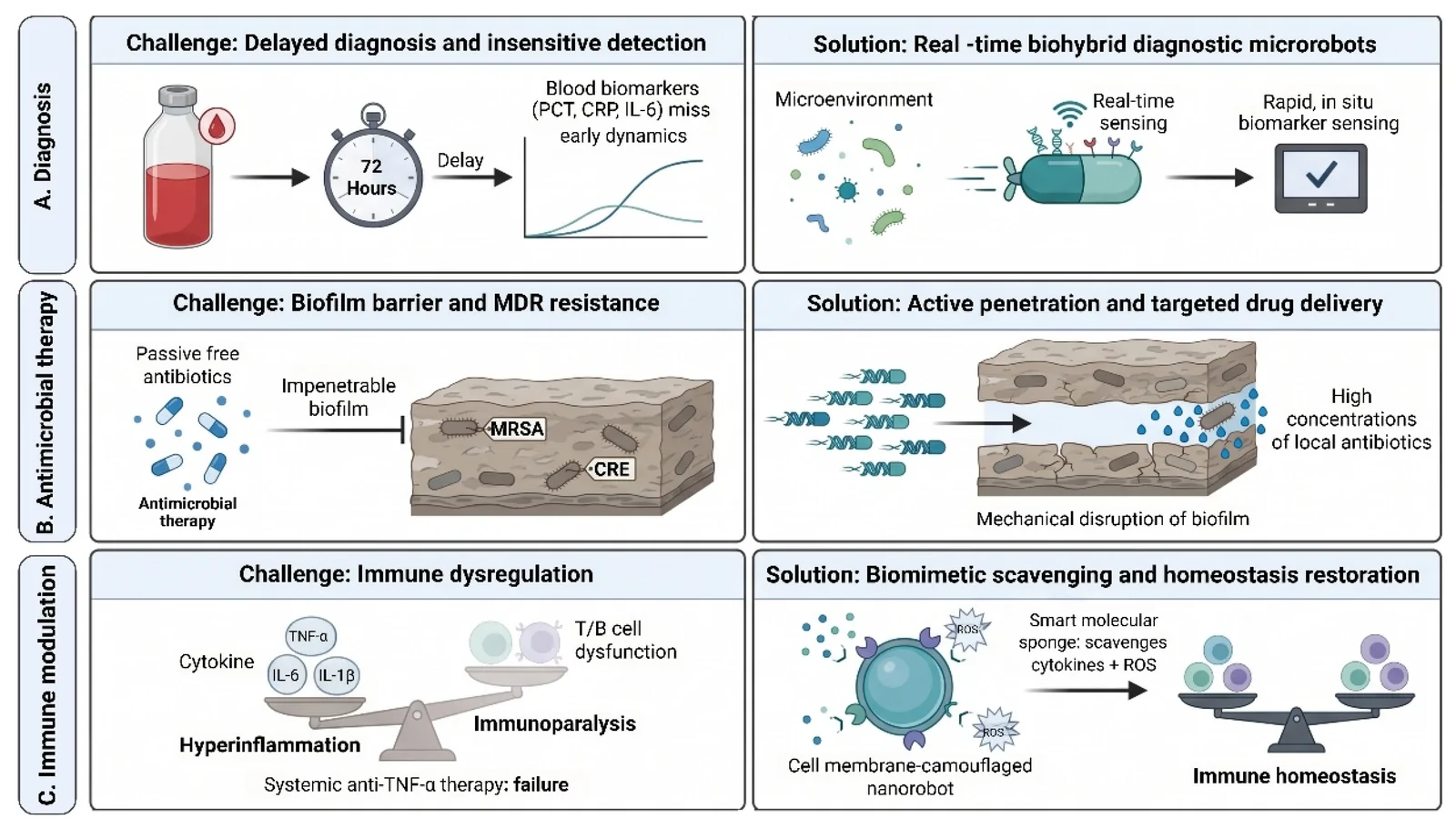
Micro/nanorobotic platforms designed as mechanism-specific solutions to address current clinical deficiencies in sepsis management. (A) Diagnosis track: Traditional diagnostics have longer turnaround times for blood culture and static laboratory processing; biohybrid microrobots functionalized with responsive DNA nanodevices or enzyme sensors can be used for rapid, real-time pathogen identification. (B) Antimicrobial therapy track: Passive free antibiotics fail to penetrate dense bacterial biofilms and combat multidrug-resistant (MDR) niches; actively propelled micro/nanorobots can use mechanical force to break down the biofilm matrix and create transport channels for enhanced localised drug accumulation. (C) Immune modulation track: Conventional systemic immunotherapies cause non-specific suppression; biomimetic nanorobots camouflaged with host cell membranes can act as molecular sponges to selectively trap circulating pro-inflammatory mediators and restore physiological homeostasis. (Created using Biorender.com).

Some research has been conducted on the pathological mechanisms of sepsis, but at present, diagnostic and therapeutic methods are not sensitive or timely enough to prevent or treat early-stage sepsis. With the rapid development and varied forms of sepsis, early detection and timely intervention in other areas are urgent problems 48, 52-54 (Figure 2).

#### Limitations of Early Diagnosis

Promptly identify sepsis to improve patient outcomes; however, at present, diagnostic tools are not very effective 55. Traditional biomarkers such as procalcitonin (PCT), C-reactive protein (CRP), and interleukin-6 (IL-6) are not ideal indicators of infection due to their slow kinetic response and lack of specificity; they only provide general information on the presence of infection and a systemic inflammatory response 56, 57. Therefore, these markers do not reflect changes in the disease at a high speed and are thus less suitable for the rapidly changing clinical situation of sepsis. New ways have been proposed to discover new biomarkers, such as multi-omics analysis (proteomics, metabolomics and transcriptomics) combined with machine learning algorithms; however, these methods are technically difficult and expensive, and their application in point-of-care testing has not yet been achieved 58-60 (Figure 2A).

#### Challenges in Antimicrobial Therapy

Antimicrobial resistance (AMR) is a serious global public health problem that has increased management difficulties for sepsis 61, 62. Clinical data show that the prevalence of multidrug-resistant (MDR) pathogens in sepsis is relatively high, such as methicillin-resistant Staphylococcus aureus (MRSA), carbapenem-resistant Enterobacteriaceae (CRE), and extensively drug-resistant Pseudomonas aeruginosa 63, 64. These strains are often resistant to the general-purpose broad-spectrum antibiotics. At the same time, indiscriminate or empirical use can increase resistance, disrupt the host microbiota, and thus raise the risk of secondary infections, such as Clostridioides difficile colitis 65-66. Phage therapy and antimicrobial peptides are other ways that have shown promise in preclinical studies but have not yet been widely applied clinically 67-68. Therefore, diagnostic and therapeutic methods that can rapidly identify pathogens and their resistance genes at the source are urgently needed (Figure 2B).

#### Challenges in Immune Modulation

The immune environment of sepsis is extremely complicated, and at the same time, hyperinflammation and immunosuppression occur simultaneously 58, 69. Immunomodulatory treatments generally fall into the following three categories. Mitigation of excessive immune response, reversal of sepsis-induced immunoparalysis (SII), and reversal of vascular tone 70. The previous ways have not worked. Anti-cytokine therapies, such as anti-TNF-α agents, have also failed to show significant improvement in clinical outcomes in trials and are likely due to non-specific suppression of protective immune functions 71. Recombinant interferon therapy (e.g., NCT03332225) is a typical immunostimulatory way employed in clinical trials that has not yielded good results. The first trials of immune checkpoint inhibitors (PD-1 and CTLA-4) have shown a potential benefit for the immune system and changes in biomarkers, and have a manageable adverse event profile in sepsis patients; however, clear clinical advantages have not been confirmed 72. Together, these results indicate that there is immune dysregulation and heterogeneity in sepsis, and a general-purpose precision immunotherapy has not yet been developed to address both hyperinflammation and impaired host defence simultaneously 70.

Given the above problems, a new type of treatment system is required to address the issues of fluctuation and multi-organ damage caused by sepsis 69-71. Promptly detect pathogens and inflammatory markers at the bedside, actively cross physiological barriers to deliver drugs to the site of infection or inflammation, and monitor for a rapid response of the body. Such features have been applied to the development of new micro- and nanorobotic systems, providing autonomous movement, biofilm penetration, targeted interaction with cells or molecules, and execution of multi-step functions; these are promising for high-precision sepsis management (Figure 2C).

## 3. Design Principles and Functionalization of Micro/Nanorobots

**Figure 3:**
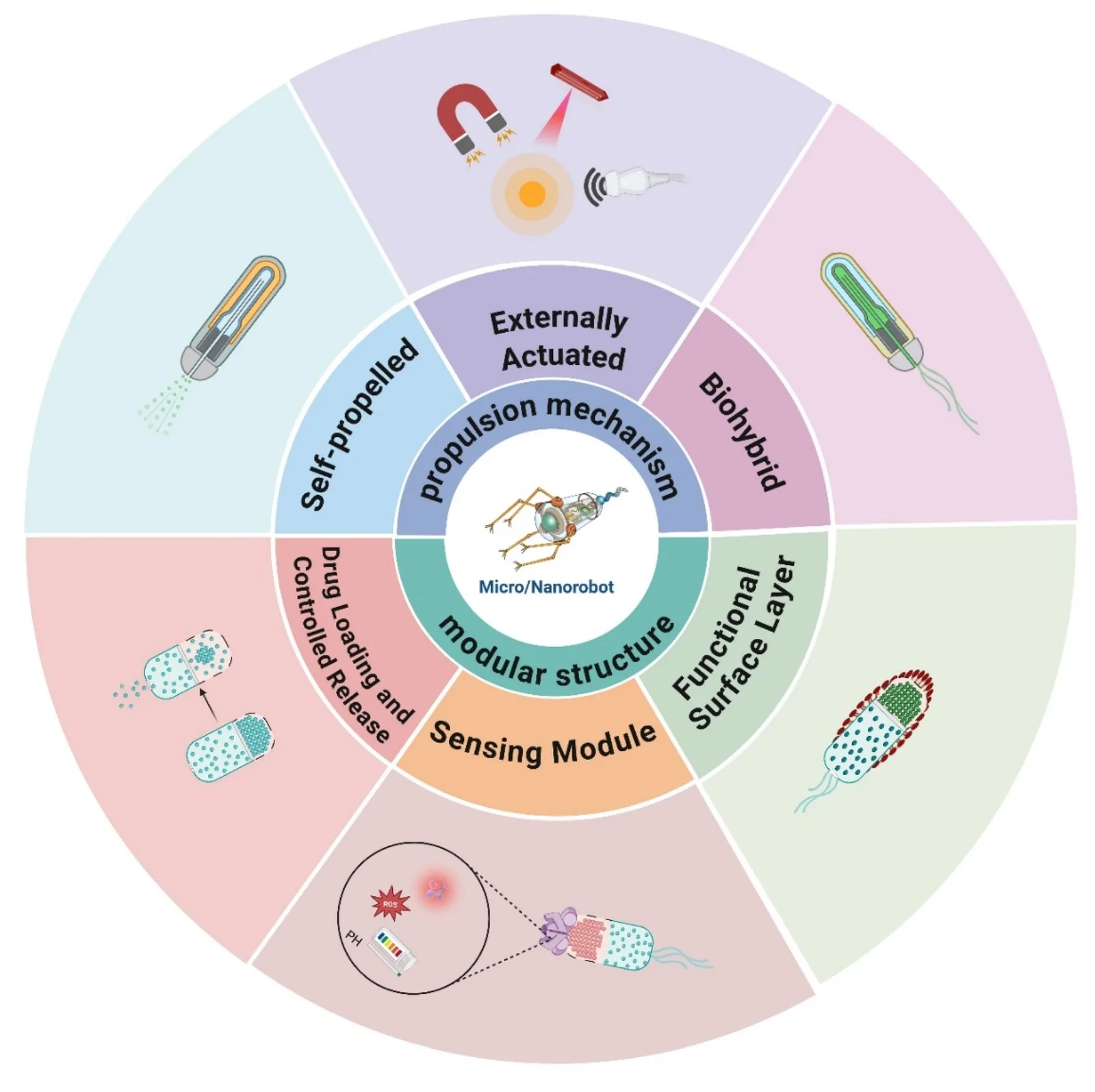
Micro/nanorobots have many types of propulsion and are modular in design for targeted biomedical applications. According to the way of propulsion, these can be classified as self-propelled (e.g., catalytic or chemical propulsion), externally actuated (e.g., magnetic, optical or acoustic control), or biohybrid systems that integrate living organisms and synthetic materials to improve both mobility and biocompatibility. A modular structure is used to achieve multi-functionality, such as drug-loading and controlled-release systems for therapeutic delivery, sensing modules for detecting environmental or pathological signals, and functional surface layers that improve biocompatibility, targeting or immune evasion. A module-and-tunable Design can be used to carry out various high-precision, flexible biomedical operations by micro- and nanobots. (Created with Biorender.com).

**Figure 4:**
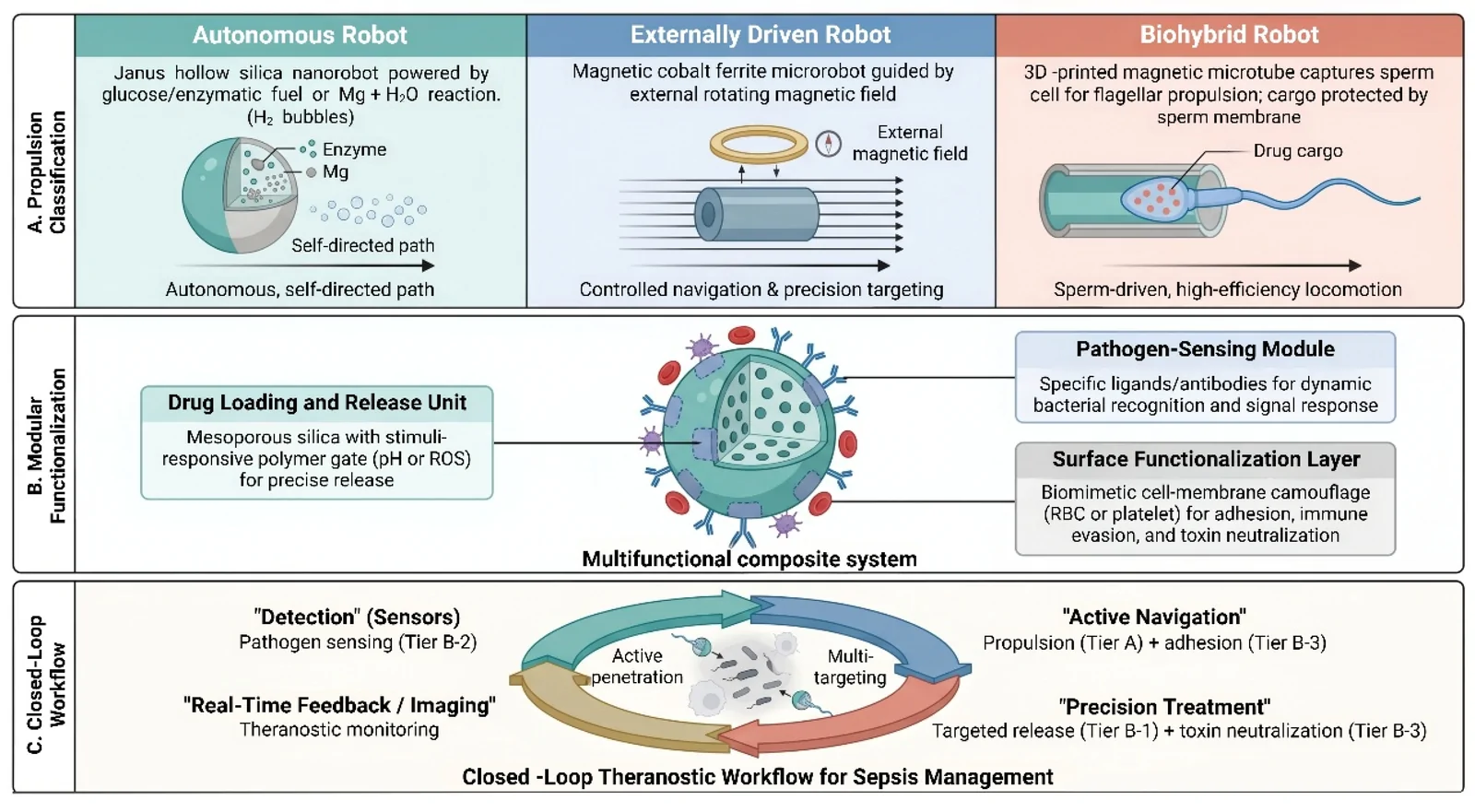
Design principles, modular functionalization and theranostic feedback of micro/nanorobots. This figure shows the basic design and operating architecture of micro- and nanorobotic systems. (A) Propulsion and Classification Tier: Micro/nanorobots are divided into autonomous, externally driven, and biohybrid systems based on endogenous biofuels, physical fields, and motile microorganisms for controlled movement. (B) Modular Functionalization Tier: Multi-functional platforms include drug-loading and stimuli-responsive release units, pathogen-sensing modules, and biomimetic surface coatings to achieve targeted delivery, dynamic recognition, and immune evasion. (C) Closed-loop Theranostic Workflow Layer: The integrated components form an all-encompassing theranostic cycle of pathogen detection, active navigation, precise treatment, and real-time feedback for intelligent and adaptive disease control. (Created with Biorender.com).

To offer strong theoretical support for this paper, some inclusion criteria will be established to distinguish between actual micro/nanobots and traditional passive nanomedicines. In our synthesis, a platform is classified as a genuine micro/nanorobot if it has kinetic autonomy; specifically, this refers to active propulsion, autonomous navigation, or externally controlled motion driven by endogenous chemical fuel or exogenous physical field 20. This mechanical self-propulsion is a significant deviation from the previous types of nanoparticles, cell membrane-coated nanomedicines, nanozymes and rigid DNA nanostructures, which are all passive Brownian diffusion or semi-active systemic blood flow transport systems 73. Importantly, although some of the aforementioned advanced platforms have incorporated cell membrane camouflage (e.g., platelet or neutrophil coatings) or catalytic nanozymes, these passive components have not been combined with the robot itself 74. At the same time, they are strictly defined as functional surface layers or therapeutic cargo attached to an active micro/nanorobotic chassis for achieving a synergistic "motility-plus-protection" feedback loop. Based on the above strict constraints, micro/nano-robots have developed highly modular architectures that combine autonomous movement with space-time precise tasks in complex biological environments (Figure 3), and they can be classified according to the strategy of propulsion and the complexity of the system. Generally speaking, the three categories of micro/nanorobots according to their driving modes are autonomous, externally driven and biohybrid systems 75-77 (Figure 4A).

(1) Autonomous micro/nanorobots are propelled by internal chemical reactions and can move autonomously without external field manipulation. These systems generally involve the reactions of active metals (e.g., magnesium or zinc) with water or acidic metabolites in physiological fluids, generating hydrogen gas to move towards infection sites for local antimicrobial action and drug delivery 78, 79. Furthermore, the inclusion of enzymatic catalytic modules allows the use of endogenous “biofuels” such as glucose or urea. For example, a biocompatible Janus hollow mesoporous silica-based nanorobot was designed to exploit the synergistic activities of catalase, urease, and glucose oxidase, enabling autonomous propulsion and sustained drug release in complex microenvironments, with the potential for dynamic management of deep-seated infections 80. (2) Externally driven micro/nanorobots rely on applied physical fields, such as magnetic, ultrasonic, or optical stimuli, for controlled navigation and precise localisation 81-83. Magnetic propulsion is typically achieved by incorporating magnetic materials (e.g., ferrite or cobalt ferrite) into the robot’s structure, enabling efficient transport to specific lesions in rotational or gradient magnetic fields, which is particularly suitable for deep-seated septic abscess clearance and drug delivery. 84, 85. Acoustic and optical propulsion systems use ultrasonic vibrations or specific wavelengths of light to induce local oscillations or photothermal effects, which induce microscale motion and trigger drug release. These exogenous stimuli-responsive strategies have the advantages of remote control and local energy concentration and are promising for targeting multi-organ microenvironmental damage and inaccessible foci of infection in sepsis 86, 87. (3) Biohybrid micro/nanorobots are a new frontier and are usually composed of motile microorganisms (e.g., bacteria or sperm) and synthetic materials to form hybrid microsystems 88. They are self-propelled by microbial movement (e.g., flagellar movement) and form biomaterials with improved biocompatibility. Such designs achieve high-efficiency locomotion and are ideal for drug delivery 89. For example, 4-armed 3D-printed magnetic microtubes have been used to capture sperm and move them using an external magnetic field. The sperm membrane contains high concentrations of drugs, protects them from dilution and enzymatic degradation, and is superior to conventional drug carriers. Alapan *et al*. similarly proposed a hybrid system integrating E. coli with magnetically encoded red blood cells, in which external magnetic fields guide their precise movement for targeted drug delivery. This design uses the motility of bacteria and the natural drug-carrying capacity of red blood cells to create a programmable and efficient delivery platform 90. Thus, biohybrid propulsion strategies hold great promise for efficient locomotion, targeted delivery, and good biocompatibility in complex physiological environments, indicating a critical pathway for the clinical translation of micro- and nanorobots.

Besides propulsion-based division, micro/nanorobots can also be categorised according to their functions in integrated single-function or multiple-function composite systems 91-92. Single-function systems are generally designed to achieve specific therapeutic goals, such as targeted drug delivery in deep tissues or physical disruption of bacterial biofilms, and are thus suitable for discrete lesion intervention. Multifunctional composite micro/nanorobots have built an all-encompassing theranostic feedback loop by combining multiple modules, such as locomotion, pathogen detection, smart cargo release and immune modulation. Modular integration is a typical approach to enhancing the therapeutic effect, and it generally consists of the three modules listed below: drug-loading and controlled-release modules, pathogen detection/signal-response units, and surface functionalisation layers.

Drug loading and release units are often constructed from porous materials (such as mesoporous silica) or microcavities in combination with stimuli-sensitive polymers that can be triggered by pH, redox conditions or reactive oxygen species (ROS) to achieve controlled and "switchable" drug release in the acidic or oxidative microenvironments of septic lesions 93-94. Thus, there will be high local drug concentrations and reduced systemic toxicity. Pathogen-sensing modules dynamically recognise pathogens (such as S. aureus) through specific ligands, antibodies or molecular probes, transmit the signal to the control unit, and activate the following therapeutic measures 95. The surface functionalisation layer directly contacts the host environment, improving biocompatibility, preventing blood clotting and inhibiting phagocytosis, and can be modified to add other pathogen-recognition and immune-regulation functions, such as biomimetic membranes, antimicrobial peptides or immunomodulatory molecules 96-97.

Esteban-Fernández de Ávila *et al*. have built ultrasound-driven biomimetic nanorobots by fusing red blood cells and platelet membranes on gold nanowires to form a hybrid biomimetic shell. This Design provided the micro/nanorobots with strong pathogen adhesion (for S. aureus) and toxin-neutralising properties (such as α-toxin antagonism). Biomimetic Nanorobots can move efficiently and continuously in whole blood samples by ultrasound. The dynamic characteristics of these biomimetic surfaces improve pathogen capture and toxin elimination, exhibiting good prospects for sepsis control 98 (Figure 4B). By means of combined operation of these three modules, micro/nanorobots can achieve a closed-loop theranostic process of "detection → navigation → treatment → feedback" in the complex septic microenvironment, providing a foundation for next-generation intelligent sepsis management (Figure 4C).

Micro/nanorobots have several advantages over the old passive-delivery system. First of all, self-propelled propulsion can cross physiological barriers and pathogen biofilms to reach deep infection sites and increase the local concentration of drugs 91, 99. Second, dual targeting via external field guidance and ligand-mediated recognition can precisely localise to reduce systemic drug exposure and off-target toxicity 100. Micro/nano-robots are also inherently theranostic devices; that is, imaging probes (e.g., MRI, photoacoustic or fluorescent tags) and therapeutic cargo can be combined on the same platform for dynamic feedback-guided therapy 101. Micro/nano-robot technology can be used to address the defects in conventional sepsis diagnosis and treatment through active penetration, bio-targeting and multi-modal integration.

## 4. Prospects for Micro/Nano-robots in Sepsis Diagnostics

Rapidly and accurately identify the pathogenic microorganisms and their secreted toxins, such as lipopolysaccharide (LPS) from gram-negative bacteria, in the early stages of sepsis to promptly initiate effective treatment 55. Conventional culture-based and biochemical assays are relatively slow, often taking several hours or even days to obtain results, and thus fail to meet the urgent demand for a "golden hour" in clinical applications. Micro- and Nano-robots: A New Path for Detection of Pathogens with High Sensitivity and Speed. Micro-/Nano-robots are inherently biosensors that can detect a wide range of biological substances by observing changes in motion or fluorescence quenching. Sanchez and others immobilised FRET-labelled triple-stranded DNA nanodevices on urease-powered mesoporous silica micromotors to create a multi-functional platform that propels and monitors the microenvironmental pH in real time. By monitoring the pH changes resulting from ammonia production during urea hydrolysis, FRET signals in the system were dynamically linked to micromotor velocity, enzyme activity and propulsion force to confirm that the DNA nanodevices functioned as sensitive reporters of micromotor activity 102. In addition, the research group has also developed a motion-driven DNA-sensing strategy that alters the catalytic velocity of nanomotors after the dissolution of silver nanoparticles (AgNPs) trapped in sandwich DNA hybridisation to achieve fast, simple and sensitive detection of DNA and bacterial rRNA 103. This motion-based biosensing method can produce a distance-readable signal, is highly sensitive and selective, and has achieved a lower limit of detection for the DNA target at 40 amol. Therefore, no separation or purification of the native bacterial rRNA is needed before the analysis. Therefore, no particle amplification is required in this nanomotor-based approach, and the risk of false signals due to non-specific silver precipitation has been reduced. In addition, it is portable, low-cost and easy to use, and thus more suitable for point-of-care testing. Although the above factors are favourable, micro- and nanorobot-based biosensors for sepsis, a serious and rapidly progressing disease, have not been widely applied; however, their diagnostic prospects are promising. Procalcitonin (PCT) is a general marker of sepsis in China that can distinguish between bacterial infection and other kinds of inflammation. Molinero-Fernández *et al*. have reported a fluorescence immunoassay platform with micro- and nanorobots for PCT detection. The three modules in this organised structure are: an outer polymeric polypyrrole (PPy) layer with a high affinity for anti-PCT antibodies, a middle nickel (Ni) layer that can be precisely externally magnetically guided, and an inner platinum nanoparticle (PtNP) core for self-propulsion via catalytic generation of oxygen bubbles. With the help of this active target recognition motility, the FMIm platform achieved an extremely low limit of detection (LOD) of 0.07 ng mL⁻¹ in a broad clinically relevant dynamic range of 0.5–150 ng mL⁻¹. Notably, this micro-robotic assay requires a very small sample volume of only 25 μL and is therefore highly suitable for screening highly vulnerable very low-birth-weight infants (VLBWIs) with suspected sepsis. Strong correlation and good statistical agreement have been verified between this micromotor-based method and the standard immunofluorescence assay in hospitals; therefore, it has been shown to be effective in practice and suitable for emergency point-of-care diagnosis of sepsis 104.

In addition, different robotic cohorts can be barcode-labeled with different fluorescent tags or surface aptamers to support multiplexed detection of multiple targets simultaneously by these mobile platforms, such as tracking PCT, IL-6 and LPS concurrently. Multi-target versatility and a lack of requirements for professional sample pre-processing or laboratory extraction equipment are needed for a high-stakes baseline of point-of-care testing (POCT) in unpredictable emergency and critical-care situations. Therefore, although current fast molecular tests such as quantitative PCR have achieved exceptional amplification systems for nucleic acids, micro/nanorobotic diagnostics are now addressing the problem of a slow turnaround time by developing unified "motion-amplified" analytical models.

## 5. Applications of Micro/Nanobots in Sepsis Therapy

Micro- and nano-robots can be used to establish a new type of sepsis treatment that actively targets pathogens by moving near them and close to them; thus, they will have improved antibacterial effects, modulate the immune system, and repair damaged tissues. To evaluate the actual translation level of these therapeutic measures and avoid promoting them prematurely in a broad clinical context, it should be noted that only robotic platforms directly validated in whole-organ sepsis models or those tested under localised and peripheral injury conditions are considered applicable at present. A true systemic sepsis model is typically one that mimics the multi-organ failure and inflammatory response of real sepsis, such as the gold-standard cecal ligation and puncture (CLP) or lethal systemic intraperitoneal bacterial challenge model. Conversely, localised platforms validated in isolated tissue environments (such as subcutaneous skin wounds, localised biofilm plaques or isolated acute lung injury models) are early-stage technologies that are only potentially relevant to sepsis treatment. Although these local systems provide basic information on biofilm disruption or cytokine scavenging, their functional parameters should be separated from systems capable of surviving the chaotic blood shear stress and multi-organ volatility of systemic septic shock.

### 5.1 Targeted Antimicrobial Therapy

**Figure 5:**
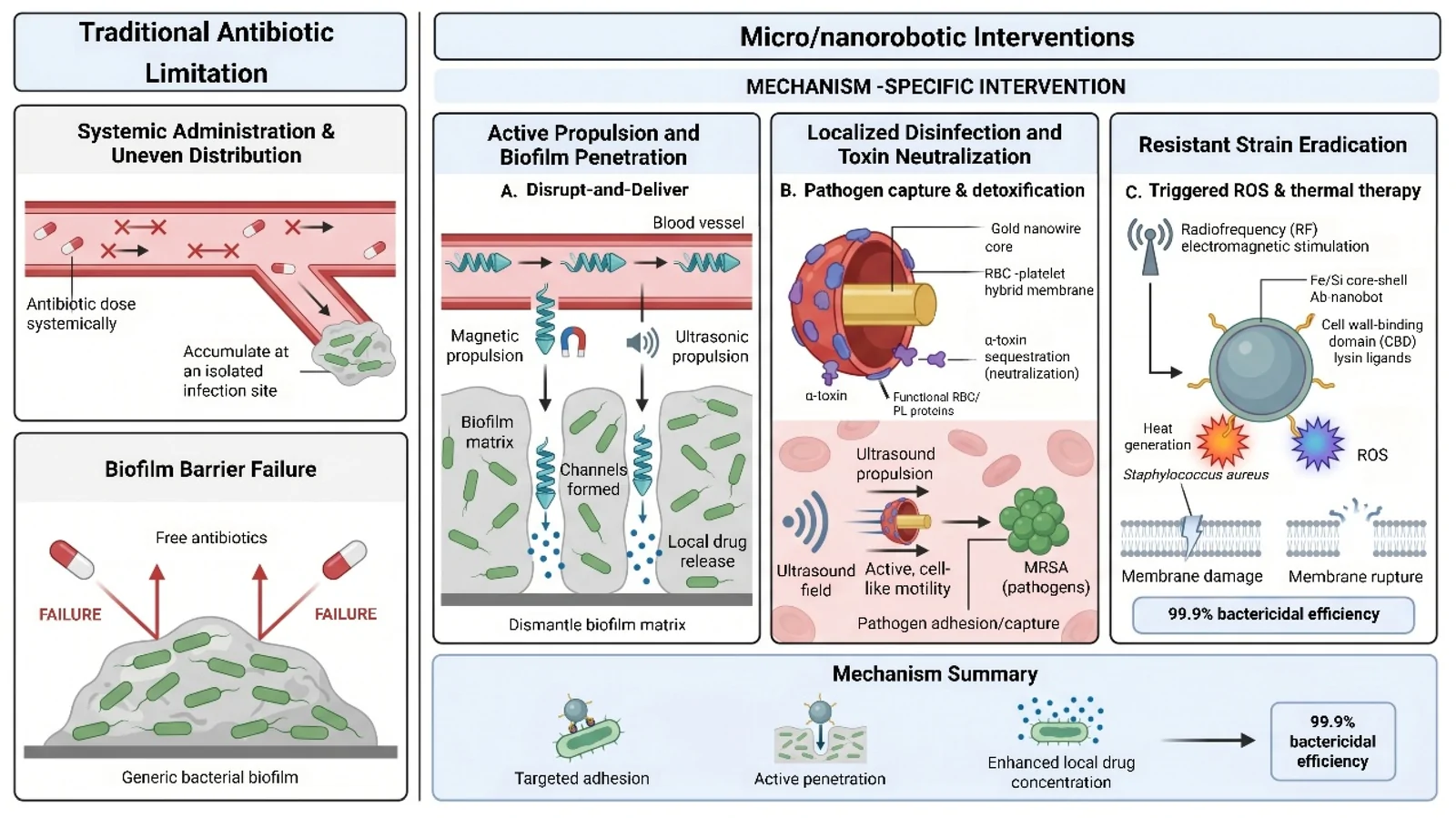
Advanced micro/nano-robotic approaches for targeted antimicrobial therapy in sepsis. Figure 1 is the new micro- and nanorobot strategies for sepsis-related infections. (A) Disruption and delivery of biofilm penetration: Due to the poor diffusion characteristics of biofilms, conventional antibiotics are ineffective; thus, actively propelled microrobots use magnetic or ultrasonic guidance to penetrate the biofilm matrix, form transport channels, and improve the local delivery of drugs. (B) Pathogen capture and detoxification: Biomimetic nanorobots with a gold nanowire core and an RBC-platelet hybrid membrane have active motility, strong bacterial adhesion, and toxin-neutralising capabilities to capture pathogens and eliminate circulating virulence factors. (C) Stimulated ROS and thermal therapy: Functionalised engineered antibacterial nanorobots specifically target pathogens such as MRSA; after external stimulation with RF radiation, they generate local heat and ROS to destroy bacteria and exhibit enhanced antimicrobial activity. (Created with Biorender.com).

Multidrug-resistant (MDR) pathogens around the world have increased, and now sepsis treatment is even more difficult. According to overseas epidemiological research, more than a quarter to more than one-third of all ICU sepsis cases worldwide have now involved multidrug-resistant (MDR) bacteria 105. Carbapenem-resistant Enterobacteriaceae (CRE) and methicillin-resistant Staphylococcus aureus (MRSA) are particular priority pathogens that frequently cause serious bloodstream infections and increase the attributable patient mortality rate to 40%-50% in the presence of septic shock 106. The above resistance data indicate that traditional empirical monotherapy has certain deficiencies in clinical application and new physical therapy methods are needed. Two main deficiencies of conventional antibiotic therapy for sepsis patients are: (i) Systemic administration results in poor distribution of drugs and low local concentrations at the site of infection, and (ii) Bacterial biofilms form a dense polymeric barrier that hinders antibiotic penetration to a certain extent 7. Given the above, micro/nanorobots, which are self-propelled and rationally designed, can break through these barriers to reach the infected site precisely for treatment. First, Chemical, magnetic, acoustic or optical propulsion can be employed to guide micro- and nanorobots to the site of infection. Ultrasound-driven biomimetic nanorobots with a gold nanowire core and a hybrid membrane of red blood cells (RBCs) and platelets (PLs) have been developed. A hybrid membrane keeps the functional proteins of RBCs and PLs to promote pathogen attachment (e.g., *Staphylococcus aureus*) and inhibit pore-forming toxins (e.g., α-toxin). These biomimetic nanorobots have exhibited rapid, efficient and persistent propulsion in whole blood under ultrasound fields without obvious contamination, and are in line with natural cell movement. Their active movement significantly enhances the efficiency of pathogen capture and detoxification, and show promise for the treatment of sepsis 98 (Figure 5A).

Secondly, biofilms are a common cause of drug-resistant bacteria because they are difficult to treat 107. They have developed biocompatible photocatalytic titanium dioxide (TiO2) microrobots for skin applications that can treat methicillin-resistant Staphylococcus aureus (MRSA). Staphylococcus aureus (MRSA) infections are subsequently coated with silver (Ag) or platinum (Pt) to enhance the photocatalytic activity and efficient self-propulsion of these microrobots under external hydrogen peroxide or ultraviolet light. Experiments show that MRSA biofilms are rapidly cleared by biomimetic membranes and pig skin tissue. Careful Dosing showed low cytotoxicity and was comparable to other clinical skin disinfectants. Histological analysis showed that effective biofilm disruption had been achieved without damage to the surrounding tissue, and thus this method was considered biocompatible and potentially therapeutic 108. (Figure 5B) It should be pointed out that this photocatalytic structure is limited to local, peripheral skin decontamination and has not been directly validated for systemic sepsis control.

Biofilm disruption and local drug delivery also provide a dual-action "disrupt-and-deliver" strategy. This way can help manage resistant strains, such as MRSA and carbapenem-resistant Enterobacteriaceae (CRE), and offers a new path for treating chronic septic infections (Figure 5C). Quan and others have designed a magnetic iron oxide nanoparticle (MIONP)-based method to actively drive MIONPs to carve microchannels in biofilms using an external magnetic field, thus dismantling dense matrices in biofilms. Confocal laser scanning microscopy showed that channels had formed and gentamicin penetrated S. aureus biofilms 4-6 times better, thus enhancing the bactericidal effect 109. Although very innovative, this "disrupt-and-deliver" model has only been validated in localised static biofilm models, and its applicability to circulating systemic blood-stream networks is unknown.

**Figure 6:**
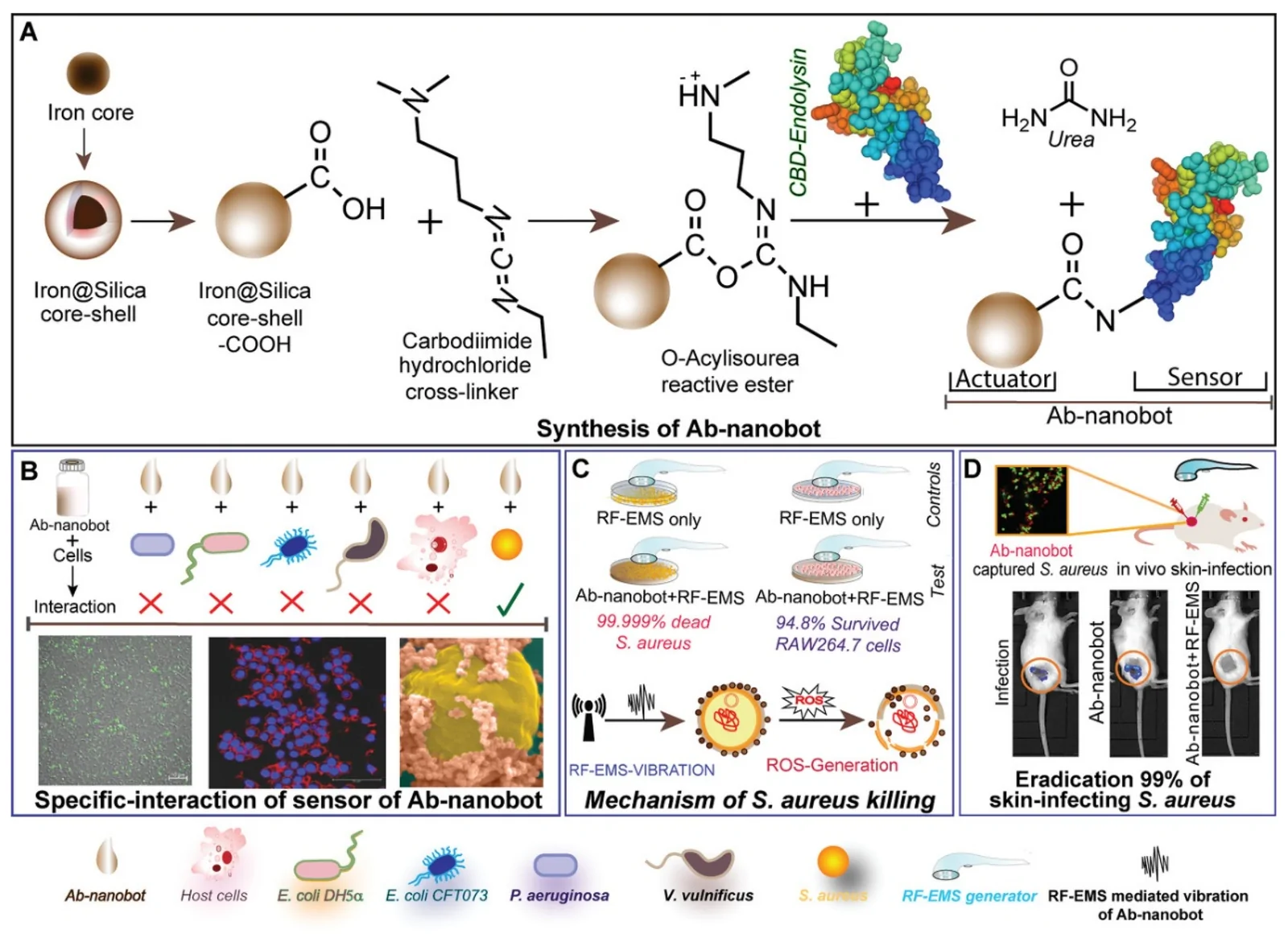
Structural configuration and functional mechanism of radiofrequency-triggered antibacterial nanorobots (Ab-nanobots) against multi-drug resistant pathogens. The active platform has been functionalised with cell-wall-binding domain lysins on a magnetic-responsive core to selectively bind to drug-resistant bacteria. Remote Radiofrequency electromagnetic stimulation generates local heat and reactive oxygen species in nanorobots to cause targeted physical damage to bacterial membranes. This bio-orthogonal physical ablation mode shows good translational potential for reducing the spread of antibiotic resistance by avoiding the biochemical pathway of multi-drug resistance evolution 110. Reproduced with permission from John Wiley and Sons (Small. 2021; 17: e2100257).

To fight against resistant pathogens, Nayab Batool and others have built an antibacterial nanorobot (Ab-nanobot) with a cell wall-binding domain (CBD) lysin that can specifically target S. Au/Fe/Si core-shell structure for heat and reactive oxygen species generation under radiofrequency electromagnetic stimulation. The system quickly and effectively reduced MRSA to a level of less than 0.01% within 20 minutes 110 (Figure 6).

Antimicrobial micromotors directly damage the cell membrane of bacteria and have also reduced the development of antibiotic resistance. For example, S can be observed and targeted precisely by the MO-1 bacterial microrobot; a magnetic-control device is installed on the robot, and after generating a concentrated, rotating and oscillating magnetic field, bacteria are effectively killed 111. The system may provide a model for the next generation of targeted antimicrobial treatment.

### 5.2 Immune Modulation and Organ Protection

**Figure 7:**
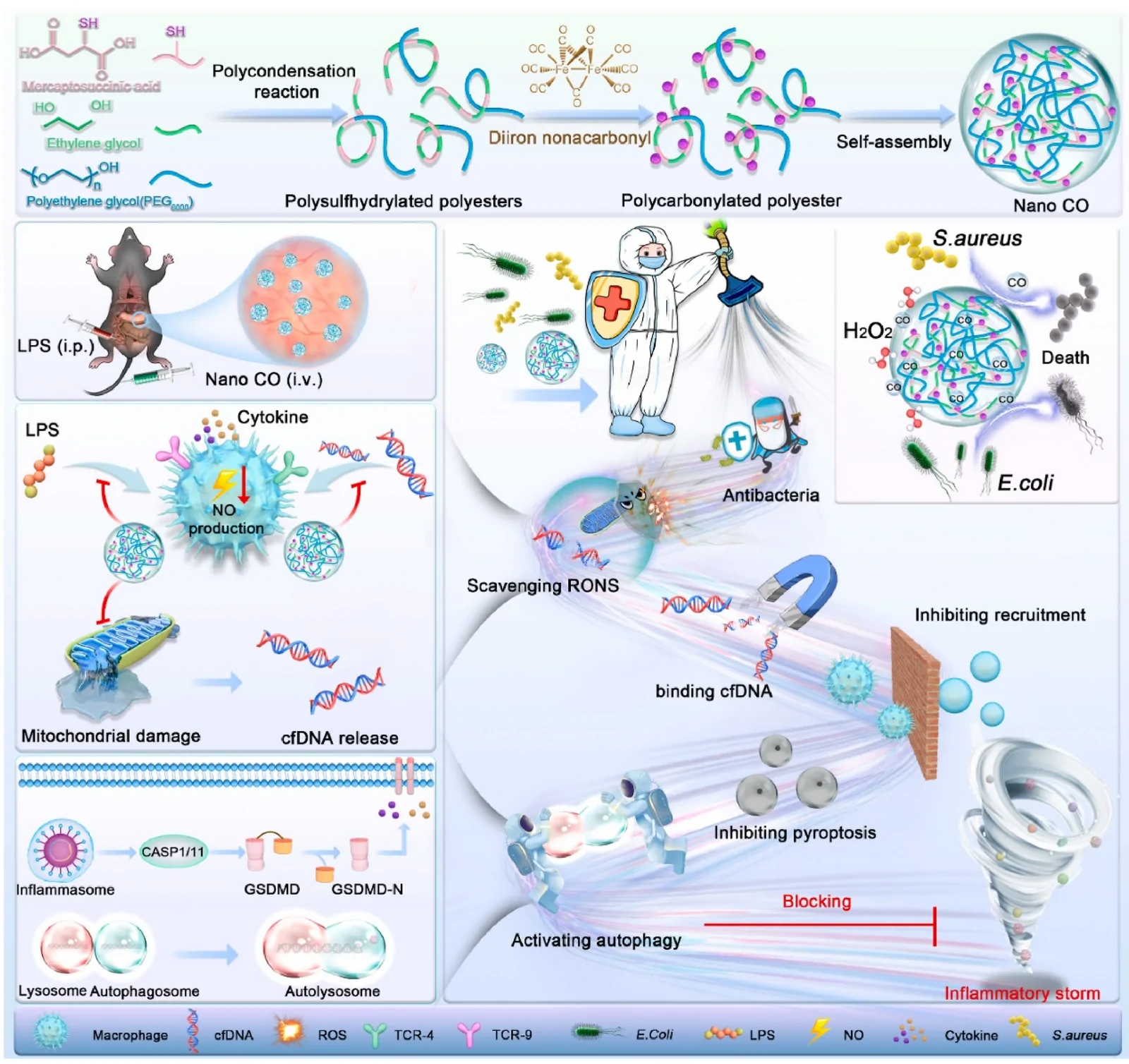
Schematic illustration of the design of self-assembled Nano-CO and its ability to scavenge multiple inflammatory mediators in sepsis. Antibacterial and inhibition of LPS-induced NO production; Scavenging of RONS (•OH, •O2⁻ and •NO) and inhibition of oxidative stress-induced DNA damage and cell death; Binding and clearance of cfDNA; Blocking activated macrophage recruitment; Inhibition of pyroptosis; Induction of autophagy 112. Reproduced with permission from Elsevier (Bioact Mater. 2024; 39: 595-611).

Sepsis is caused by both the increased number of pathogens and a failure of the host immune system; it begins with an excessive release of pro-inflammatory cytokines (cytokine storm) and may later lead to immunosuppression. Wu *et al*. have developed a self-assembled multi-functional carbon monoxide nanogenerator (Nano CO) in the early hyperinflammatory stage. Nano CO is exposed to reactive oxygen species (ROS) and thus releases CO; it has antibacterial and immunomodulatory properties. Nano Co activates the heme oxygenase-1/CO signaling pathway to enhance the body's own defence mechanisms by reducing ROS and cell-free DNA (cfDNA), inhibiting macrophage activation and pyroptosis, and promoting autophagy. Research on animals has shown that LPS-induced sepsis can be reduced in severity, damaged organs may be repaired, and about 50% fewer deaths occur 112 (Figure 7).

Chen and others also coated Fe3O4 nanoparticles with neutrophil membranes to create a magnetically guided nanorobot (MAGICIAN) that retained the function of the membrane. MAGICIAN migrated to the liver under a magnetic field, and *in vitro* it reduced inflammatory cytokines (IL-6, TNF-α, IFN-γ) to alleviate inflammation in an acute lung injury mouse model 113.

**Figure 8:**
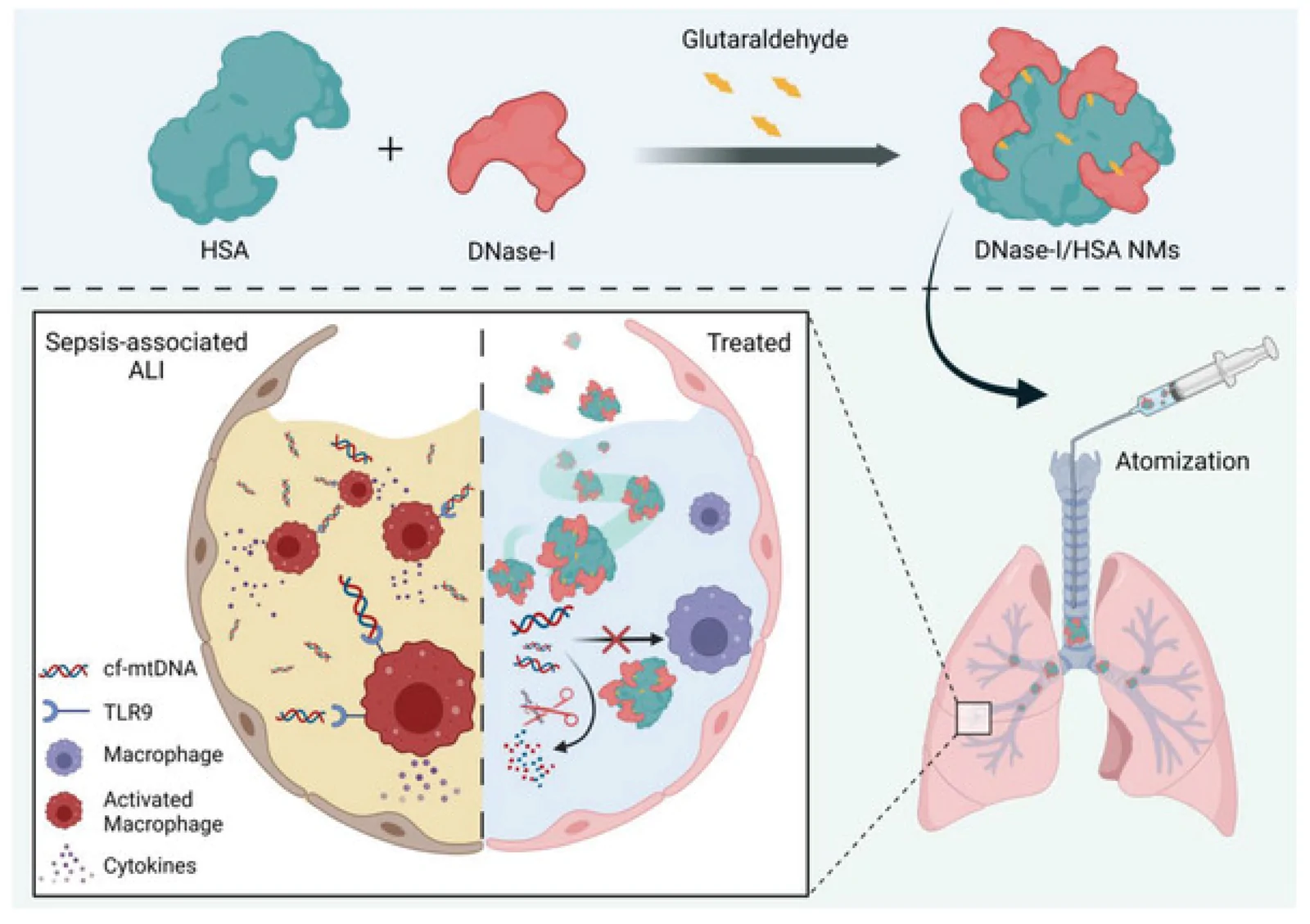
Synthetic route of DNase-I/HSA NMs and their application in treating sepsis-associated ALI. Synthesis of DNase-I/HSA NMs by glutaraldehyde-mediated crosslinking of HSA and DNase-I (upper panel). Self-propulsive DNase-I/HSA NMs efficiently scavenged cf-mtDNA in the pulmonary microenvironment and thus effectively blocked cf-mtDNA/TLR9-mediated alveolar macrophage activation, alleviating sepsis-associated ALI upon aerosolized intratracheal instillation (lower panel) 114. Reproduced with permission from John Wiley and Sons (Adv Sci (Weinh). 2023; 10: e2301635).

Circulating mitochondrial DNA (circ-mtDNA) can be the cause of pulmonary inflammation and injury in sepsis. Biocompatible hybrid protein nanomotors were prepared by Huang and others through glutaraldehyde crosslinking of DNase I and human serum albumin (HSA). This inhalable platform is self-propelling; it hydrolyzes cf-mtDNA in the lungs, reduces pulmonary inflammation and tissue damage, and improves survival in septic models 114. (Figure 8) Although this targeted neutralisation is a significant advance in tissue-specific acute organ damage, its clinical efficacy has not been fully verified at the level of the peripheral lung microenvironment and further studies, such as systemic lethal sepsis challenges, are needed to determine full translational readiness.

Zhou *et al*. Designed a DNA-based nanorobot that was functionalized with the Ac-PGP peptide to specifically bind to CXCR2 on neutrophils and target the recruitment of neutrophils to sites of inflammation. This tetrahedral DNA nanorobot (APT) extends the half-life of neutrophils, regulates their life cycle and maturation, and effectively inhibits oxidative stress, inflammation and neutrophil migration; thus, it attenuates tissue damage caused by sepsis 115. For the purpose of meeting the dual requirements of antibacterial and anti-inflammatory properties, Song *et al*. designed a biodegradable magnesium micromotor loaded with tobramycin (Mg-Tob motor) as a hydrogen generator and active antibiotic delivery vehicle. Mg can continuously react with water in the peritoneal environment of septic mice to produce hydrogen gas and thus drive the motor and reduce hyperinflammation. The system was antibacterial and anti-inflammatory in animals; it did not cause multi-organ damage and increased the survival rate of severe sepsis to 87.5% 113.

Together, the above platforms have shown that the essential engineering characteristics should be adjusted based on specific pathological conditions. The performance trade-offs of these systems are different due to various factors, such as the propulsion mode, driving mechanism, scale of application and material composition, etc. For example, although micrometer-sized, autonomous chemical-driven motors have generated a strong driving force suitable for penetrating impenetrable biofilm matrices via the intraperitoneal route; however, their gas-bubble generation can lead to local hyperalkalynisation and vascular embolism under severe systemic septic conditions. Nanoscale external-field driven or bioresponsive DNA devices offer safer safety evaluations and precise cargo delivery in systemic cecal ligation models, but their active targeting strategies are often limited by circulating nucleases or strong reticuloendothelial system sequestration. Therefore, based on the evaluation of therapeutic effects across different routes of administration and disease models, it has been determined that no single design is universally applicable; thus, the design rule for localised disinfection and systemic immunomodulation should be established to provide translational insights for overcoming the existing technical barriers in precision sepsis therapy (Table 1).

## 6. Challenges and Future Directions

**Figure 10:**
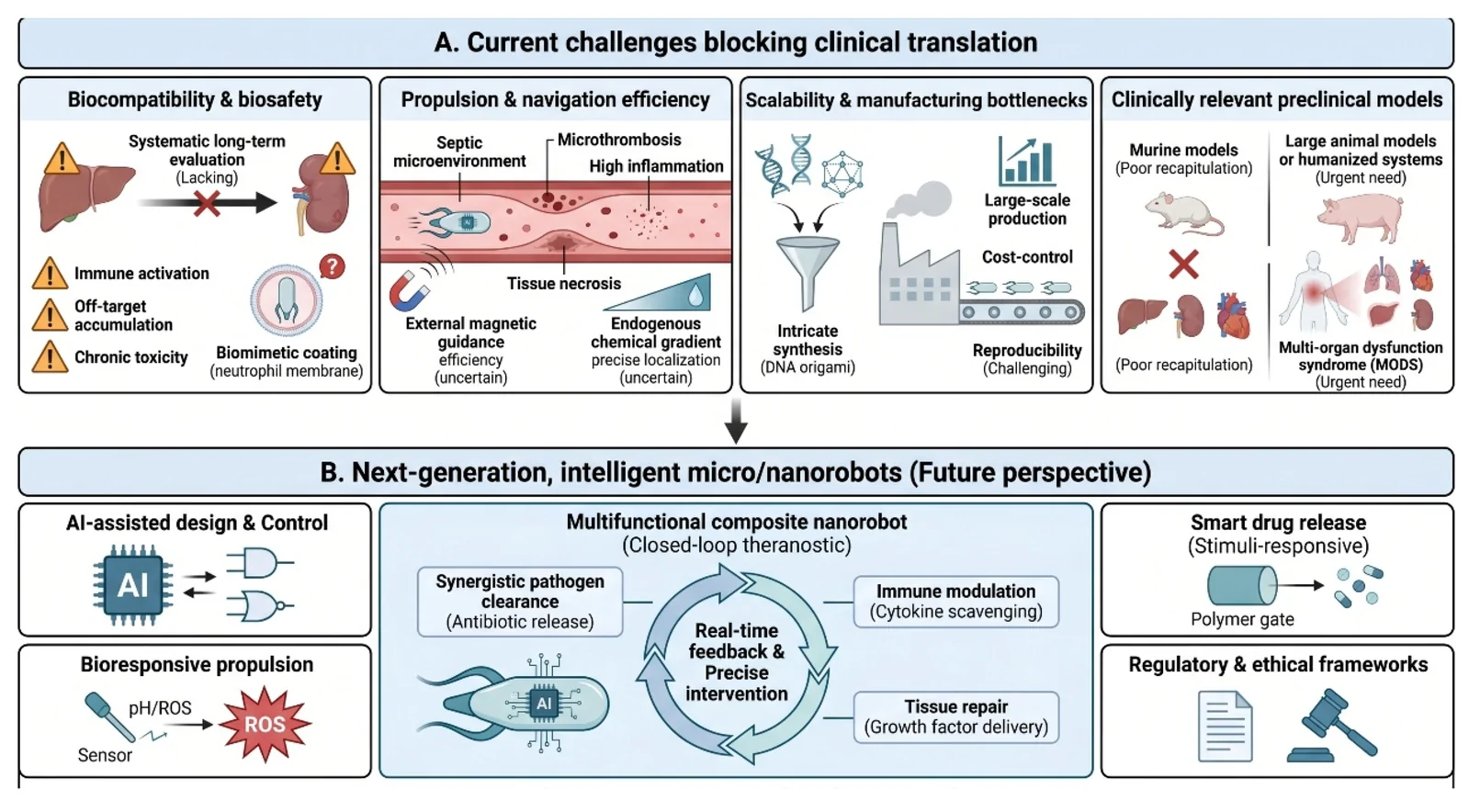
Current clinical translational challenges and future prospects of sepsis micro-/nano-robots. This figure summarizes the main problems in the clinical translation and future directions for sepsis micro- and nanorobotics. (A) Present Challenges: (i) Biocompatibility and long-term biosafety; (ii) Suboptimal propulsion and navigation efficiency in complex septic microenvironments; (iii) Problems with scalable and reproducible manufacturing; (iv) Lack of clinically relevant preclinical models that can accurately replicate multi-organ dysfunction in humans are hindering clinical translation. (B) Looking Ahead: We expect that next-generation intelligent micro/nanorobots will be closed-loop therapeutic platforms equipped with AI-assisted design, bioresponsive propulsion and stimuli-responsive drug delivery to achieve coordinated pathogen elimination, immune modulation and tissue repair under a safe and clinically regulated framework. (Created with Biorender.com).

Micro- and nanorobots have shown some promise in sepsis treatment, but many problems still need to be solved before they can be used clinically (Figure 10A). Biomimetic Coatings, such as neutrophil membranes and human serum albumin, have been used to enhance immune evasion and reduce toxicity; however, their long-term safety data in humans are unavailable. An evaluation of the present preclinical data shows that although these active-propulsion synthetic micro/nanorobots have been developed, most are still easily captured by the reticuloendothelial system (RES) and accumulate excessively off-target in the liver and spleen 116. The increased accumulation of hepatic and splenic macrophages thus reduces the systemic circulation half-life of the active vectors and may lead to prolonged histocompatibility issues due to chronic intracellular retention of metallic or polymeric degradation by-products 117. Therefore, an organized study of nanoparticle degradation kinetics and chronic organ clearance pathways under normal disease conditions is required but has not been carried out. Second, the propulsion and navigation in a complex and dynamic *in vivo* environment are not yet effective. Most of the current micro/nanorobots are externally controlled by physical fields (magnetic, acoustic, etc.) or internal chemical gradients for navigation, and their navigation efficiency in the context of sepsis is not well known; sepsis involves high inflammation, microthrombosis and tissue necrosis. Additionally, the unusual autonomous and biohybrid structures present particular biosafety risks and require higher safety margins. Magnesium-based autonomous micromotors can convert chemical energy into motion efficiently, but they may also be more susceptible to degradation in acidic septic lesions; thus, there is a risk of localised hyperalkalisation and damaging hydrogen gas bubble accumulation within the restricted vascular bed 118. At the same time, enzyme-powered systems and living biohybrid platforms (such as flagellated bacterial hybrids) are also highly immunogenic; their synthetic enzymes or foreign cellular components may trigger severe, counterproductive secondary inflammatory reactions that worsen the pre-existing host cytokine storm 119. Third, there are still technical problems of scale-up and production. Many designs of micro/nanorobots involve complex synthesis and assembly processes (e.g., multifunctional composite structures and DNA origami robots), which are not suitable for large-scale production, cost control and batch-to-batch reproducibility required by clinical application. Fourth, there is a strong demand for more all-encompassing and clinically applicable preclinical models. Most of the current studies use mice as models, and these do not reflect the heterogeneity of human immune responses or the complexity of multi-organ dysfunction in sepsis. Large-animal models and humanised systems can be used to assess the effectiveness and safety of a drug in a more clinical way. Fourth, the problem of regulation and industrial production barriers to clinical translation has yet to be solved. Scaling up production has created a serious quality control bottleneck in maintaining batch-to-batch consistency, and for biohybrid platforms, small biological variations can alter the surface ligand density and thus cause uneven *in vivo* biodistribution. In addition, the usual sterilization methods for pharmaceuticals (autoclaving or gamma irradiation) may cause damage to fragile surface-targeting proteins or destroy camouflage membranes, and therefore cannot be used to extend the functional shelf life of these materials in emergency clinical practice. Crucially, because these active platforms simultaneously contain synthetic materials, biological ligands and drug payloads, they fall under the strict and ambiguous jurisdiction of "combination products" according to the US FDA and EMA guidelines. Navigation through the many regulatory review channels lacks a specific evaluation system for autonomous robots and is thus a significant translation bottleneck.

Fifth, given the serious physiological instability of sepsis patients, more all-encompassing preclinical models are urgently required. In the turbulent circulatory system, *in vivo* propulsion needs to overcome high hemodynamic shear stress without damaging the endothelium, and introducing synthetic metallic or polymeric cores into a deregulated immune microenvironment may trigger microthrombosis or enhance disseminated intravascular coagulation (DIC). These platforms should also be able to work well with the emergency clinical workflow and quickly resume standard resuscitation procedures. Most of the current research uses mice, and these models do not well replicate the volatility of human diseases, immune heterogeneity and multi-organ dysfunction. Therefore, extended toxicity studies and the construction of large-animal models or humanised systems are required to evaluate the safety and efficacy of septic shock in a realistic setting. In the future, the next generation of micro/nanorobots will be multi-functional modules for synergistic pathogen elimination, immune system regulation and tissue repair, and they will work with real-time feedback mechanisms for precise intervention (Figure 10B). Artificial Intelligence-assisted Design and control, bioresponsive propulsion and smart drug-release systems will be introduced to improve the efficacy and safety of therapy. Regulations and ethical norms for applying autonomous nanosystems in serious illness will also need to be established to guide their clinical application. In spite of the above problems, micro/nano-robots are generally ready to promote sepsis treatment through specific, active, and personalised ways, and have provided new hope for the global medical crisis of sepsis.

## 7. Summary

**Figure 11:**
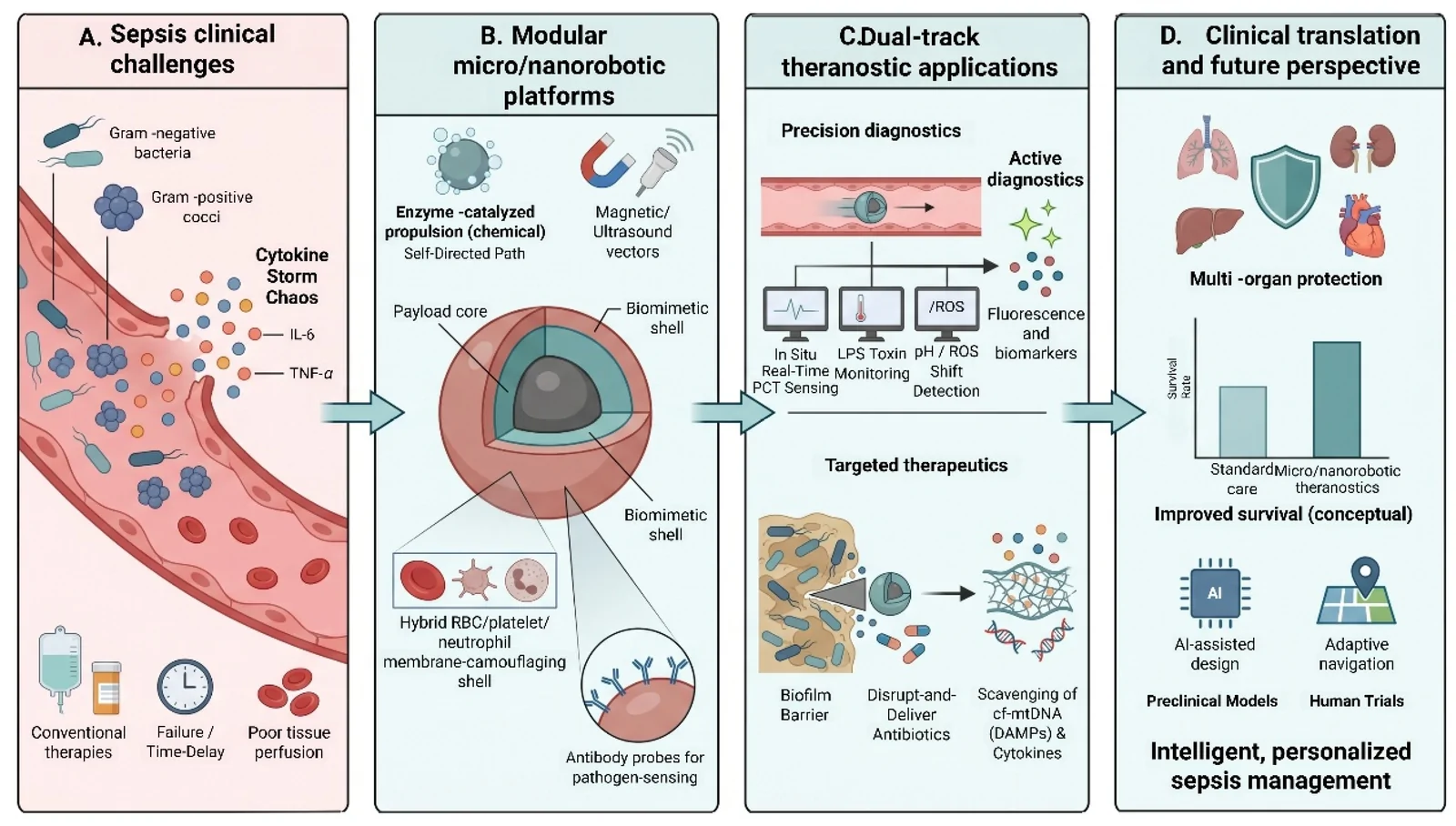
Integrated micro/nano-robotic platform for smart and personalised sepsis management. We propose an integrated framework for micro- and nanorobots in the treatment of sepsis. (A) Clinical problems of sepsis: Traditional static antibiotic therapy is constrained by the early stage of infection, cytokine storm and vascular damage. (B) Modular micro/nanorobotic platforms: The designed nanorobots have incorporated propulsion systems and biomimetic functional coatings for targeted navigation and biological compatibility. (C) Dual-track theranostic applications: These platforms can perform high-precision diagnosis by in situ sensing of biomarkers and changes in the microenvironment, and simultaneously conduct targeted therapy, such as active biofilm disruption and inflammatory mediator scavenging. (D) Clinical translation and future prospects: AI-assisted navigation and intelligent feedback systems will eventually achieve personalised intervention, multi-organ protection and restoration of systemic homeostasis. (Created with Biorender.com).

Micro- and nanorobots as integrated multifunctional therapeutic platforms have shown exceptional promise for sepsis treatment. These systems address some deficiencies in the traditional methods of drug delivery and immune modulation for tissue repair by using autonomous propulsion, precise navigation, multiple drug-loading capabilities and biomimetic designs. Recently, studies have shown that micro- and nanorobots can be employed to disperse biofilms, deliver drugs precisely, reduce cytokine storms and regulate the immune system, protect organs, etc. They are new ways to deal with the problems of sepsis. Problems such as biosafety, long-term stability, *in vivo* dynamics and scalable manufacturing still need to be solved, but with continuous progress in materials science, fabrication technology and intelligent-responsive systems, clinical application will be promoted. In the future, micro/nanorobots that can be integrated with artificial intelligence will be able to perform full-course intervention of sepsis management in a personalised manner based on early diagnosis, such as *in vivo* monitoring and multimodal therapy (Figure 11).

## Figures and Tables

**Figure 9:**
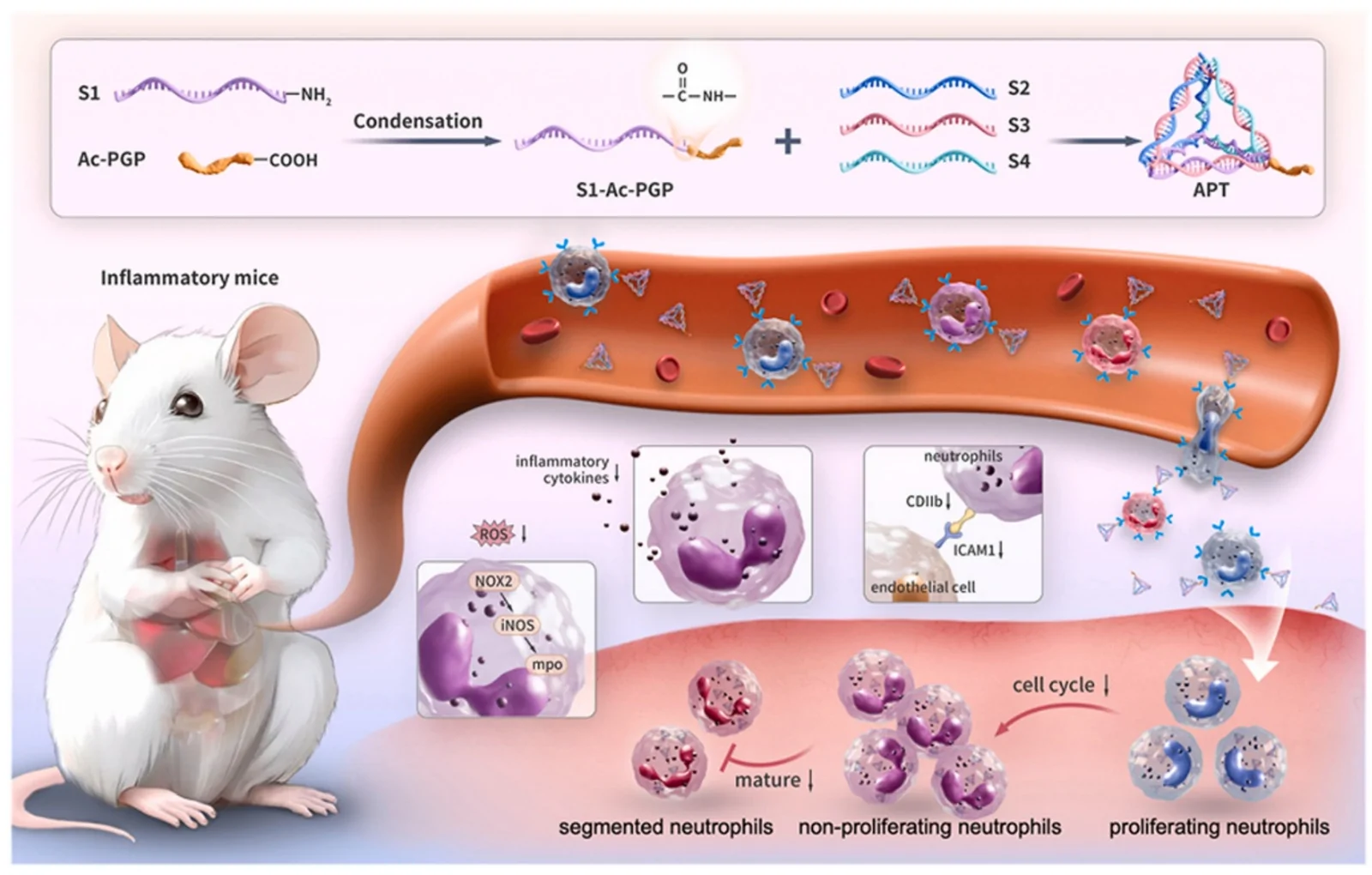
Schematic illustration of the role of APT nanorobots in targeting, hitchhiking and regulating neutrophils for enhanced sepsis therapy. A DNA-based nanorobot was designed to target neutrophils with an N-acetyl Pro-Gly-Pro (Ac-PGP) peptide for specific binding to the C-X-C motif of chemokine receptor 2 (CXCR2) on the membrane of neutrophils. The robot (tetrahedral framework nucleic acid modified with Ac-PGP, APT) recognised and hitchhiked neutrophils to accumulate at inflammatory sites and extended its half-life. The robot can also be used to inhibit the division and maturation of neutrophil cells, regulate oxidative stress and inflammation, and suppress migration and recruitment in both *in vivo* and *in vitro* inflammation experiments 115. Reproduced with permission from Elsevier (Biomaterials. 2025; 318: 123183).

**Table 1:** Comparison of representative micro/nano-robotic platforms for sepsis therapy

Robotic platform	Propulsion mechanism	Major therapeutic target	Experimental model	Main advantages	Major limitations	Most suitable septic stage	Ref.
RBC/PL hybrid membrane-coated biomimetic nanorobot	Ultrasound-driven	Pathogen capture and toxin neutralization	Whole blood / bacterial infection	Efficient propulsion in blood, biomimetic immune evasion, simultaneous pathogen adsorption and toxin neutralization	Requires external ultrasound guidance; systemic efficacy requires further validation	Early bloodstream infection	95
TiO₂ photocatalytic microrobot (Ag/Pt-coated)	Photocatalytic (UV/H₂O₂)	MRSA biofilm eradication	Skin biofilm / pig skin	Excellent biofilm penetration and bacterial eradication	Applicable only to localized infections; unsuitable for systemic circulation	Localized soft-tissue infection	103
Magnetic iron oxide nanoparticles (MIONPs)	Magnetic field-driven	Biofilm disruption and antibiotic delivery	S. aureus biofilm	Creates microchannels to enhance antibiotic penetration (4–6-fold increase)	Validated only in static biofilm models	Localized biofilm-associated infection	104
Antibacterial nanorobot (Ab-nanobot)	Radiofrequency-triggered	MRSA killing	*In vitro* MRSA	Extremely rapid antibacterial activity (99.99% killing within 20 min)	Lack of systemic sepsis validation	Drug-resistant bacterial infection	105
MO-1 bacteria-guided microrobot	Magnetic propulsion	Physical bacterial membrane disruption	Microfluidic platform	Precise magnetic navigation with reduced likelihood of resistance	Prototype system; no *in vivo* sepsis evaluation	Precision antimicrobial therapy (proof-of-concept)	106
Nano CO self-assembled nanogenerator	ROS-responsive self-activation	Cytokine storm suppression, ROS scavenging, autophagy induction	LPS-induced sepsis	Simultaneously antibacterial, anti-inflammatory and organ-protective; reduced mortality	Mainly effective during hyperinflammatory phase	Early hyperinflammatory stage	107
MAGICIAN (neutrophil membrane-coated Fe₃O₄ nanorobot)	Magnetic targeting	Cytokine adsorption and immune modulation	Acute lung injury	Biomimetic targeting with efficient cytokine neutralization	Organ-specific rather than systemic treatment	Inflammation-associated organ injury	108
DNase I/HSA hybrid protein nanomotor	Self-propelled	cf-mtDNA degradation	Septic lung injury	Targeted degradation of inflammatory cf-mtDNA with lung protection	Limited systemic applicability	Sepsis-induced lung injury	109
Ac-PGP-functionalized DNA nanorobot (APT)	Ligand-mediated targeting	Neutrophil regulation	Sepsis model	Precisely regulates neutrophil recruitment and oxidative stress	DNA nanostructures remain susceptible to nuclease degradation	Transition from hyperinflammation to immune regulation	110
Tobramycin-loaded biodegradable Mg micromotor (Mg-Tob motor)	Hydrogen-powered chemical propulsion	Dual antibacterial and anti-inflammatory therapy	CLP-induced sepsis	Combines active drug delivery with hydrogen-mediated immunomodulation; improved survival (87.5%)	Hydrogen generation may complicate clinical translation	Severe systemic sepsis	113

## Data Availability

The data supporting the results of this study are available from the corresponding author upon reasonable request.
